# Identification of flavonolignans from *Silybum marianum* seeds as allosteric protein tyrosine phosphatase 1B inhibitors

**DOI:** 10.1080/14756366.2018.1497020

**Published:** 2018-08-30

**Authors:** Ningbo Qin, Tatsunori Sasaki, Wei Li, Jian Wang, Xiangyu Zhang, Dahong Li, Zhanlin Li, Maosheng Cheng, Huiming Hua, Kazuo Koike

**Affiliations:** a Key Laboratory of Structure-Based Drug Design & Discovery, Ministry of Education, Shenyang Pharmaceutical University, Shenyang, Liaoning, PR China;; b School of Traditional Chinese Materia Medica, Shenyang Pharmaceutical University, Shenyang, LiaoningPR China;; c Faculty of Pharmaceutical Sciences, Toho University, Funabashi, Japan;; d School of Pharmaceutical Engineering, Shenyang Pharmaceutical University, Shenyang, Liaoning, PR China

**Keywords:** Protein tyrosine phosphatase 1B, flavonolignan, silybin, isosilybin, *Silybum marianum*

## Abstract

Protein tyrosine phosphatase 1B (PTP1B) is an attractive molecular target for anti-diabetes, anti-obesity, and anti-cancer drug development. From the seeds of *Silybum marianum*, nine flavonolignans, namely, silybins A, B (**1**, **2**), isosilybins A, B (**3**, **4**), silychristins A, B (**5**, **6**), isosilychristin A (**7**), dehydrosilychristin A (**8**), and silydianin (**11**) were identified as a novel class of natural PTP1B inhibitors (IC_50_ 1.3 7–23.87 µM). Analysis of structure–activity relationship suggested that the absolute configurations at C-7" and C-8" greatly affected the PTP1B inhibitory activity. Compounds **1**–**5** were demonstrated to be non-competitive inhibitors of PTP1B based on kinetic analyses. Molecular docking simulations resulted that **1**–**5** docked into the allosteric site, including α3, α6, and α7 helix of PTP1B. At a concentration inhibiting PTP1B completely, compounds **1**–**5** moderately inhibited VHR and SHP-2, and weakly inhibited TCPTP and SHP-1. These results suggested the potentiality of these PTP1B inhibitors as lead compounds for further drug developments.

## Graphical Abstract

**Figure:**
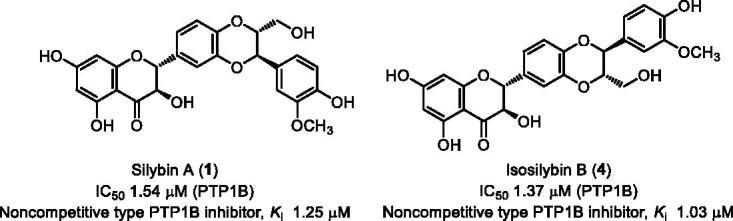

## Introduction

Protein tyrosine phosphatase 1B (PTP1B) is a non-transmembrane protein tyrosine phosphatase^1^, expressing ubiquitously in the classical insulin-targeted tissues, and negatively regulating insulin and leptin signalling pathway^2^. Inhibition of PTP1B has been demonstrated beneficial effects, such as increased insulin sensitivity, improved glucose tolerance, and resistance to high-fat-induced weight gain, but without side effects^3^. Thus, PTP1B inhibitors have attracted much attention for anti-diabetes and anti-obesity drug developments^4^. Recently, PTP1B also found a positive role in the tumorigenesis of breast cancer and colorectal cancer, extending the application of PTP1B inhibitors as anti-cancer agents^5^. Up to date, several PTP1B inhibitors, such as ertiprotafib^6^, and trodusquemine^7^, have been developed into clinical trials. Despite the fact that chemical synthetic PTP1B inhibitors have reached to quite potent inhibitory activities, some barriers are remaining, such as low PTP selectivity, low bioavailability, and insufficient *in vivo* efficacy^8^. In contrast, natural products are recognised as another important resource to discover novel PTP1B inhibitors^9^.

*Silybum marianum* (L.) Gaertn. (Asteraceae), also known as milk thistle, is a well-known medicinal plant both in Europe and Asia^10^. *S. marianum* has been reported to have various pharmacological effects, such as hepatoprotective, anti-inflammatory, antifibrotic, and antioxidant effects^11–13^. Recently, the effectiveness of flavanolignans from the seeds of *S. marianum* in ameliorating diabetes either *in vitro* or *in vivo* was also reported^14–17^.

During our ongoing investigations to discover novel PTP1B inhibitors from natural resources, we have reported a number of natural PTP1B inhibitors^18–22^, which included canthinone alkaloids from Simaroubaceae medicinal plants, isoprenylated flavonoids from *Glycyrrhiza* species and *Sophora flavescens*, neolignans from *Magnolia officinalis*, anthraquinones from *Rheum officinale*, and triterpenes and neolignans from *Sambucus adnate*. These natural PTP1B inhibitors shared a great structural diversity, which was different from synthetic inhibitors, providing more chance for development of novel PTP1B inhibitors. Herein, we report the identification of flavanolignans from the seeds of *S. marianum* as a novel class of natural allosteric PTP1B inhibitors.

## Materials and methods

### Instruments and chemicals

Optical rotation was measured with a Rudolph Autopol-V digital polarimeter (Rudolph Research Analytical Co., New Jersey, USA) or a Perkin Elmer Model 341 polarimeter (PerkinElmer Inc., Massachusetts, USA). NMR spectra were recorded on a Bruker ARX-400 or an AV-600 NMR spectrometer (Bruker Co., Rheinstetten, Germany) with tetramethylsilane (TMS) as an internal standard. Semi-preparative HPLC was composed of a Shimadzu LC-6AD pump system equipped with an SPD-20A PDA detector (Shimadzu Co., Ltd., Kyoto, Japan) and a C_18_ column (250 mm ×10 mm, 5 µm; YMC-ODS-A). Column chromatography (CC) was performed with silica gel (100–200 and 200–300 mesh; Qingdao Marine Chemical Ltd., Qingdao, China), ODS (S-50 µm; YMC Co., Ltd., Kyoto, Japan), Sephadex LH-20 (GE Healthcare Biosciences AB, Uppsala, Sweden), and polyamine (Qingdao Marine Chemical Ltd., Qingdao, China). The absorbance in PTP1B bioassays was measured and recorded on a 2300 EnSpire Multimode Plate Reader (PerkinElmer, Hamburg, Germany). The chemical reagents were as follows. PTP1B (human recombinant), T-cell protein tyrosine phosphatase (TCPTP, human recombinant), and Vaccinia H1-related phosphatase (VHR, human recombinant) were from Enzo Life Sciences, Inc. (Lausen, Switzerland). Src homology domain 2-containing protein tyrosine phosphatase 1 and 2 (SHP-1 and SHP-2, human recombinant), ursolic acid (purity >98%), citrate buffer solution (pH 6.0), *para*-nitrophenylphosphate (*p*-NPP), and bovine serum albumin were from Sigma-Aldrich Co., LLC. (St Louis, MO). Sodium chloride (NaCl), dithiothreitol (DTT), and sodium hydroxide (NaOH) were from Wako Pure Chemical Industries, Ltd. (Osaka, Japan), and ethylenediaminetetraacetic acid (EDTA) was from Dojindo Co., Ltd. (Kumamoto, Japan).

### Plant material

The seeds of *S. marianum* (L.) Gaertn. were purchased from Liaoning Shengbo Pharmaceutical Co., Ltd. (Shenyang, China), and identified by Professor Jincai Lu (Shenyang Pharmaceutical University). A voucher specimen (SM2014) has been deposited at the Department of Traditional Chinese Materia Medica, Shenyang Pharmaceutical University, China.

### Extraction and bioassay-guided isolation

The seeds of *S. marianum* (30 kg) were powdered and defatted by extraction with petroleum ether. The residue was extracted with 95% ethanol (60 L) under reflux for three times. After filtration and evaporation, a crude extract (2.0 kg) (PTP1B inhibition rate: 73.1% at 1.0 mg/mL) was obtained. The crude extract was suspended in water and sequentially partitioned with CH_2_Cl_2_, EtOAc, and *n*-butanol to yield CH_2_Cl_2_ (184 g), EtOAc (200 g), and *n*-butanol soluble fraction (390 g). The EtOAc fraction was chosen for further separation because it exhibited more potent PTP1B inhibitory activity (inhibition rate: 92.1% at 1.0 mg/mL) than CH_2_Cl_2_, *n*-butanol, and water fractions (inhibition rate: 62.0%, 49.3%, and 59.2% at 1.0 mg/mL, respectively). The EtOAc extract was fractionated by silica gel CC, eluting with gradient CH_2_Cl_2_–MeOH (from 1:0 to 0:1, *v*/*v*), to afford seven fractions (A–G). Among them, fractions E and G exhibited the most potent inhibitory activity (inhibition rate: 97.8% and 91.2% at 1.0 mg/mL).

Fraction E (10.0 g) was subjected to an ODS column, and eluted with a step gradient of MeOH–H_2_O (25:75, 35:65, 45:55, 55:45, 70:30, 100:0) to afford six fractions. Further separation of fraction E-4 (1.5 g) by RP-HPLC with MeOH–H_2_O (47:53, *v*/*v*) afforded compounds **9** (5.2 mg) and **10** (6.1 mg). Separation of fraction E-6 (2.3 g) by RP-HPLC with MeOH–H_2_O (52:48, *v*/*v*) afforded **1** (15.0 mg), **2** (16.2 mg), **3** (17.3 mg), and **4** (12.5 mg).

Fraction G (130.0 g) was fractionated by silica gel CC, using a gradient of CH_2_Cl_2_–MeOH (from 1:0 to 0:1, *v*/*v*), to give five fractions, G1–G5. Fraction G1 (0.9 g) was further separated by polyamine CC, eluting with a step gradient of MeOH–H_2_O (20:80, 30:70, 40:60, 50:50, 70:30, 100:0) and combined into two fractions, G1-1 and G1-2. Fraction G1-2 (0.12 g) was recrystallised using MeOH to afford **11** (24.7 mg). Fraction G2 (74.0 g) was fractionated by ODS CC, eluting with a step gradient of MeOH–H_2_O (30:70, 40:60, 45:55, 50:50, 55:45, 60:40, 100:0, *v*/*v*), and combined into two fractions, G2-1 (25 g) and G2-2 (3.0 g). Fraction G2-1 was separated by RP-HPLC with MeOH–H_2_O (45:55, *v*/*v*) to afford **5** (25.8 mg), **6** (20.4 mg), and **7** (4.8 mg), respectively. Fraction G2-2 was separated by RP-HPLC with MeOH–H_2_O (58:42, *v*/*v*) to yield **8** (4.7 mg).

### Silybin A (1)

Colourless solid. ${\left[\alpha \right]}_{D}^{20}$ +23.1 (*c* 0.78, acetone). ^1^H NMR (DMSO-d_6_, 400 MHz) *δ* 11.93 (1H, br s, 5-OH), 7.08 (1H, d, *J* = 1.7 Hz, H-2'), 7.01 (1H, d, *J* = 1.6 Hz, H-2''), 7.00 (1H, dd, *J* = 8.3, 1.7 Hz, H-6'), 6.96 (1H, d, *J* = 8.3 Hz, H-5'), 6.86 (1H, dd, *J* = 8.2, 1.6 Hz, H-6''), 6.80 (1H, d, *J* = 8.2 Hz, H-5''), 5.86 (1H, d, *J* = 1.9 Hz, H-8), 5.81 (1H, d, *J* = 1.9 Hz, H-6), 5.05 (1H, d, *J* = 11.2 Hz, H-2), 4.91 (1H, d, *J* = 7.9 Hz, H-7''), 4.58 (1H, d, *J* = 11.2 Hz, H-3), 4.17 (1H, m, H-8''), 3.78 (3H, s, 3''-OCH_3_), 3.54 (1H, dd, *J* = 12.5, 2.2 Hz, H-9"a), 3.34 (1H, dd, *J* = 12.5, 4.8 Hz, H-9"b). ^13^ C NMR (DMSO-d_6_, 100 MHz) *δ* 82.5 (C-2), 71.3 (C-3), 197.2 (C-4), 163.3 (C-5), 95.3 (C-6), 168.3 (C-7), 96.3 (C-8), 162.4 (C-9), 100.0 (C-10), 130.2 (C-1'), 116.5 (C-2'), 143.6 (C-3'), 143.2 (C-4'), 116.3 (C-5'), 121.3 (C-6'), 127.5 (C-1"), 111.7 (C-2"), 147.6 (C-3"), 147.0 (C-4"), 115.3 (C-5"), 120.5 (C-6"), 75.8 (C-7"), 78.1 (C-8"), 60.2 (C-9"), 55.7 (3"-OCH_3_).

### Silybin B (2)

Colourless solid. ${\left[\alpha \right]}_{D}^{20}$ –6.2 (*c* 0.87, acetone). ^1^H NMR (DMSO-d_6_, 600 MHz) *δ* 11.94 (1H, s, 5-OH), 7.07 (1H, d, *J* = 1.3 Hz, H-2'), 7.01 (1H, d, *J* = 1.3 Hz, H-2''), 7.00 (1H, br s, H-6'), 6.97 (1H, s, H-5'), 6.86 (1H, dd, *J* = 7.9, 1.3 Hz, H-6''), 6.80 (1H, d, *J* = 7.9 Hz, H-5''), 5.84 (1H, s, H-8), 5.80 (1H, s, H-6), 5.04 (1H, d, *J* = 11.3 Hz, H-2), 4.90 (1H, d, *J* = 7.9 Hz, H-7''), 4.55 (1H, d, *J* = 11.3 Hz, H-3), 4.15 (1H, m, H-8''), 3.78 (3H, s, 3''-OCH_3_), 3.54 (1H, m, H-9"a), 3.34 (1H, dd, *J* = 12.1, 4.7 Hz, H-9"b). ^13^ C NMR (DMSO-d_6_, 150 MHz) *δ* 82.5 (C-2), 71.4 (C-3), 197.3 (C-4), 163.3 (C-5), 95.3 (C-6), 167.9 (C-7), 96.3 (C-8), 162.4 (C-9), 100.1 (C-10), 130.2 (C-1'), 116.6 (C-2'), 143.6 (C-3'), 143.2 (C-4'), 116.3 (C-5'), 121.1 (C-6'), 127.5 (C-1"), 111.6 (C-2"), 147.6 (C-3"), 147.0 (C-4"), 115.3 (C-5"), 120.5 (C-6"), 75.8 (C-7"), 78.1 (C-8"), 60.2 (C-9"), 55.7 (3"-OCH_3_).

### Isosilybin A (3)

Colourless solid. ${\left[\alpha \right]}_{D}^{20}$ +32.6 (*c* 0.82, acetone). ^1^H NMR (DMSO-d_6_, 600 MHz) *δ* 11.90 (1H, s, 5-OH), 10.83 (1H, s, 7-OH), 9.13 (1H, s, 4"-OH), 7.09 (1H, d, *J* = 1.7 Hz, H-2'), 7.00 (1H, d, *J* = 1.8 Hz, H-2''), 6.98 (1H, dd, *J* = 8.3, 1.7 Hz, H-6'), 6.93 (1H, d, *J* = 8.3 Hz, H-5'), 6.85 (1H, dd, *J* = 8.0, 1.8 Hz, H-6''), 6.80 (1H, d, *J* = 8.0 Hz, H-5''), 5.92 (1H, d, *J* = 2.0 Hz, H-8), 5.88 (1H, d, *J* = 2.0 Hz, H-6), 5.10 (1H, d, *J* = 11.2 Hz, H-2), 4.91 (1H, d, *J* = 7.9 Hz, H-7''), 4.59 (1H, d, *J* = 11.2 Hz, H-3), 4.16 (1H, m, H-8''), 3.78 (3H, s, 3''-OCH_3_), 3.53 (1H, dd, *J* = 12.2, 2.0 Hz, H-9"a), 3.34 (1H, dd, *J* = 12.2, 4.6 Hz, H-9"b). ^13^ C NMR (DMSO-d_6_, 150 MHz) *δ* 82.5 (C-2), 71.5 (C-3), 197.7 (C-4), 163.3 (C-5), 95.0 (C-6), 166.8 (C-7), 96.0 (C-8), 162.5 (C-9), 100.5 (C-10), 130.3 (C-1'), 116.4 (C-2'), 143.9 (C-3'), 142.9 (C-4'), 116.4 (C-5'), 120.9 (C-6'), 127.5 (C-1"), 111.7 (C-2"), 147.6 (C-3"), 146.9 (C-4"), 115.3 (C-5"), 120.4 (C-6"), 75.8 (C-7"), 78.0 (C-8"), 60.2 (C-9"), 55.7 (3"-OCH_3_).

### Isosilybin B (4)

Colourless solid. ${\left[\alpha \right]}_{D}^{20}$ –48.9 (*c* 0.84, acetone). ^1^H NMR (DMSO-d_6_, 400 MHz) *δ* 11.92 (1H, br s, 5-OH), 7.09 (1H, d, *J* = 1.7 Hz, H-2'), 7.00 (1H, d, *J* = 1.5 Hz, H-2''), 6.98 (1H, dd, *J* = 8.3, 1.7 Hz, H-6'), 6.93 (1H, d, *J* = 8.3 Hz, H-5'), 6.86 (1H, dd, *J* = 8.2, 1.5 Hz, H-6''), 6.80 (1H, d, *J* = 8.2 Hz, H-5''), 5.87 (1H, d, *J* = 1.8 Hz, H-8), 5.84 (1H, d, *J* = 1.8 Hz, H-6), 5.08 (1H, d, *J* = 11.1 Hz, H-2), 4.92 (1H, d, *J* = 7.8 Hz, H-7''), 4.57 (1H, d, *J* = 11.1 Hz, H-3), 4.16 (1H, m, H-8''), 3.78 (3H, s, 3''-OCH_3_), 3.54 (1H, dd, *J* = 12.2, 2.2 Hz, H-9"a), 3.34 (1H, dd, *J* = 12.2, 4.6 Hz, H-9"b). ^13^ C NMR (DMSO-d_6_, 100 MHz) *δ* 82.4 (C-2), 71.4 (C-3), 197.2 (C-4), 163.4 (C-5), 95.3 (C-6), 168.2 (C-7), 96.3 (C-8), 162.4 (C-9), 100.1 (C-10), 130.4 (C-1'), 116.4 (C-2'), 143.8 (C-3'), 142.9 (C-4'), 116.4 (C-5'), 120.9 (C-6'), 127.5 (C-1"), 111.7 (C-2"), 147.6 (C-3"), 147.0 (C-4"), 115.3 (C-5"), 120.4 (C-6"), 75.8 (C-7"), 78.0 (C-8"), 60.2 (C-9"), 55.7 (3"-OCH_3_).

### Silychristin A (5)

Colourless solid. ${\left[\alpha \right]}_{D}^{20}$ +55.4 (*c* 1.03, acetone). ^1^H NMR (DMSO-d_6_, 400 MHz) *δ* 11.91 (1H, s, 5-OH), 10.84 (1H, s, 7-OH), 9.34 (1H, s, 3'-OH), 9.03 (1H, s, 4"-OH), 6.96 (1H, d, *J* = 1.7 Hz, H-2''), 6.87 (1H, br s, H-6'), 6.82 (1H, d, *J* = 1.2 Hz, H-2'), 6.81 (1H, dd, *J* = 8.2, 1.7 Hz, H-6''), 6.72 (1H, d, *J* = 8.2 Hz, H-5''), 5.91 (1H, d, *J* = 2.0 Hz, H-8), 5.86 (1H, d, *J* = 2.0 Hz, H-6), 5.76 (1H, d, *J* = 6.2 Hz, 3-OH), 5.46 (1H, d, *J* = 6.9 Hz, H-7"), 4.99 (1H, d, *J* = 11.4 Hz, H-2), 4.52 (1H, dd, *J* = 11.4, 6.2 Hz, H-3), 3.76 (3H, s, 3''-OCH_3_), 3.71 (1H, m, H-9"a), 3.63 (1H, m, H-9"b), 3.47 (1H, dd, *J* = 12.7, 6.9 Hz, H-8''). ^13^ C NMR (DMSO-d_6_, 100 MHz) *δ* 83.3 (C-2), 71.7 (C-3), 197.8 (C-4), 163.3 (C-5), 95.0 (C-6), 166.9 (C-7), 96.1 (C-8), 162.6 (C-9), 100.4 (C-10), 130.0 (C-1'), 115.6 (C-2'), 140.8 (C-3'), 147.1 (C-4'), 129.1 (C-5'), 115.4 (C-6'), 132.4 (C-1"), 110.4 (C-2"), 147.6 (C-3"), 146.4 (C-4"), 115.3 (C-5"), 118.7 (C-6"), 87.1 (C-7"), 53.4 (C-8"), 63.0 (C-9"), 55.7 (3"-OCH_3_).

### Silychristin B (6)

Colourless solid. ${\left[\alpha \right]}_{D}^{20}$ –57.9 (*c* 0.91, acetone). ^1^H NMR (DMSO-d_6_, 400 MHz) *δ* 11.90 (1H, s, 5-OH), 9.34 (2H, br s, 3'-OH, 4"-OH), 6.97 (1H, br s, H-2''), 6.91 (1H, br s, H-6'), 6.81 (2H, br s, H-2', H-6''), 6.77 (1H, d, *J* = 8.1 Hz, H-5''), 5.88 (1H, d, *J* = 1.3 Hz, H-8), 5.84 (1H, d, *J* = 1.3 Hz, H-6), 5.47 (1H, d, *J* = 6.8 Hz, H-7"), 4.99 (1H, d, *J* = 11.4 Hz, H-2), 4.54 (1H, d, *J* = 11.4 Hz, H-3), 3.77 (3H, s, 3''-OCH_3_), 3.72 (1H, m, H-9"a), 3.64 (1H, m, H-9"b), 3.47 (1H, dd, *J* = 12.4, 6.8 Hz, H-8''). ^13^ C NMR (DMSO-d_6_, 100 MHz) *δ* 83.2 (C-2), 71.5 (C-3), 197.6 (C-4), 163.4 (C-5), 95.3 (C-6), 167.7 (C-7), 96.3 (C-8), 162.6 (C-9), 100.2 (C-10), 130.0 (C-1'), 116.3 (C-2'), 140.7 (C-3'), 147.1 (C-4'), 129.3 (C-5'), 115.4 (C-6'), 132.5 (C-1"), 110.4 (C-2"), 147.6 (C-3"), 146.4 (C-4"), 115.2 (C-5"), 118.7 (C-6"), 87.1 (C-7"), 53.4 (C-8"), 63.1 (C-9"), 55.7 (3"-OCH_3_).

### Isosilychristin A (7)

Colourless solid. ${\left[\alpha \right]}_{D}^{20}$ +119.5 (*c* 1.78, acetone). ^1^H NMR (DMSO-d_6_, 600 MHz) *δ* 7.06 (1H, d, *J* = 8.3 Hz, H-6'), 6.92 (1H, d, *J* = 1.7 Hz, H-2''), 6.83 (1H, d, *J* = 8.3 Hz, H-5'), 6.82 (1H, dd, *J* = 8.2, 1.7 Hz, H-6''), 6.71 (1H, d, *J* = 8.2 Hz, H-5''), 5.93 (1H, d, *J* = 2.0 Hz, H-8), 5.91 (1H, d, *J* = 2.0 Hz, H-6), 5.67 (1H, d, *J* = 2.5 Hz, H-7"), 5.17 (1H, d, *J* = 11.8 Hz, H-2), 4.62 (1H, d, *J* = 11.8 Hz, H-3), 3.89 (1H, dd, *J* = 11.0, 4.3 Hz, H-9"a), 3.67 (1H, dd, *J* = 11.0, 9.1 Hz, H-9"b), 3.76 (4H, s, H-8'', 3''-OCH_3_). ^13^ C NMR (DMSO-d_6_, 150 MHz) *δ* 81.6 (C-2), 73.3 (C-3), 198.5 (C-4), 164.3 (C-5), 96.3 (C-6), 168.6 (C-7), 97.4 (C-8), 165.2 (C-9), 101.9 (C-10), 126.3 (C-1'), 129.6 (C-2'), 147.9 (C-3'), 142.9 (C-4'), 117.5 (C-5'), 120.8 (C-6'), 134.9 (C-1"), 110.4 (C-2"), 148.9 (C-3"), 147.3 (C-4"), 115.9 (C-5"), 119.7 (C-6"), 88.7 (C-7"), 54.2 (C-8"), 65.2 (C-9"), 56.3 (3"-OCH_3_).

### Dehydrosilychristin A (8)

Colourless solid. ${\left[\alpha \right]}_{D}^{20}$ +56.1 (*c* 0.54, acetone). ^1^H NMR (DMSO-d_6_, 400 MHz) *δ* 7.70 (1H, br s, H-2'), 7.69 (1H, br s, H-6'), 7.01 (1H, d, *J* = 1.8 Hz, H-2''), 6.88 (1H, dd, *J* = 8.1, 1.8 Hz, H-6''), 6.79 (1H, d, *J* = 8.1 Hz, H-5''), 6.37 (1H, br s, H-8), 6.16 (1H, d, *J* = 1.6 Hz, H-6), 5.62 (1H, d, *J* = 6.2 Hz, H-7"), 3.89 (2H, m, H_2_-9"), 3.58 (1H, m, H-8''), 3.83 (3H, s, 3''-OCH_3_). ^13^ C NMR (DMSO-d_6_, 100 MHz) *δ* 147.8 (C-2), 137.3 (C-3), 177.3 (C-4), 162.5 (C-5), 99.3 (C-6), 165.8 (C-7), 94.5 (C-8), 158.2 (C-9), 104.5 (C-10), 125.9 (C-1'), 117.2 (C-2'), 142.2 (C-3'), 150.3 (C-4'), 130.4 (C-5'), 117.1 (C-6'), 134.4 (C-1"), 110.6 (C-2"), 149.1 (C-3"), 147.6 (C-4"), 116.2 (C-5"), 119.8 (C-6"), 89.6 (C-7"), 55.3 (C-8"), 64.8 (C-9"), 56.4 (3"-OCH_3_).

### Silydianin (9)

Colourless solid. ${\left[\alpha \right]}_{D}^{20}$ +166.3 (*c* 0.10, MeOH). ^1^H NMR (DMSO-d_6_, 400 MHz) *δ* 11.81 (1H, s, 5-OH), 10.91 (1H, s, 7-OH), 8.79 (1H, s, 4"-OH), 7.18 (1H, s, 3'-OH), 6.75 (1H, d, *J* = 1.6 Hz, H-2''), 6.64 (1H, d, *J* = 8.1 Hz, H-5''), 6.55 (1H, dd, *J* = 8.1, 1.6 Hz, H-6''), 6.02 (1H, d, *J* = 6.4 Hz, H-6'), 5.93 (1H, d, *J* = 5.8 Hz, 3-OH), 5.91 (1H, d, *J* = 1.9 Hz, H-8), 5.89 (1H, d, *J* = 1.9 Hz, H-6), 4.86 (1H, d, *J* = 10.4 Hz, H-2), 4.44 (1H, d, *J* = 10.4, 5.8 Hz, H-3), 4.12 (1H, m, H-9"a), 3.78 (1H, d, *J* = 7.9 Hz, H-9"b), 3.74 (3H, s, 3''-OCH_3_), 3.45 (1H, dd, *J* = 3.8, 2.0 Hz, H-2'), 3.31 (1H, br s, H-7"), 3.18 (1H, dd, *J* = 8.5, 2.8 Hz, H-5'), 2.73 (1H, br s, H-8''). ^13^ C NMR (DMSO-d_6_, 100 MHz) *δ* 81.7 (C-2), 70.8 (C-3), 196.5 (C-4), 163.4 (C-5), 95.0 (C-6), 166.9 (C-7), 96.2 (C-8), 162.0 (C-9), 100.3 (C-10), 139.5 (C-1'), 48.6 (C-2'), 96.7 (C-3'), 201.9 (C-4'), 53.4 (C-5'), 124.0 (C-6'), 133.0 (C-1"), 112.4 (C-2"), 147.2 (C-3"), 145.1 (C-4"), 114.9 (C-5"), 120.3 (C-6"), 46.0 (C-7"), 44.0 (C-8"), 72.8 (C-9"), 55.4 (3"-OCH_3_).

### Dehydrosilydianin (10)

Colourless solid. ${\left[\alpha \right]}_{D}^{20}$ –44.0 (*c* 0.45, MeOH). ^1^H NMR (DMSO-d_6_, 400 MHz) *δ* 12.33 (1H, s, 5-OH), 8.79 (1H, s, 4"-OH), 7.27 (1H, br s, 3'-OH), 6.97 (1H, dd, *J* = 7.0, 2.0 Hz, H-6'), 6.68 (1H, d, *J* = 1.9 Hz, H-2''), 6.62 (1H, d, *J* = 8.2 Hz, H-5''), 6.51 (1H, dd, *J* = 8.2, 1.9 Hz, H-6''), 6.40 (1H, d, *J* = 2.0 Hz, H-8), 6.20 (1H, d, *J* = 2.0 Hz, H-6), 4.23 (1H, dd, *J* = 8.0, 3.2 Hz, H-9"a), 4.18 (1H, dd, *J* = 4.1, 2.2 Hz, H-2'), 3.86 (1H, d, *J* = 8.0 Hz, H-9"b), 3.64 (3H, s, 3''-OCH_3_), 3.46 (1H, dd, *J* = 7.0, 2.8 Hz, H-5'), 3.41 (1H, br s, H-7"), 2.81 (1H, br s, H-8"). ^13^ C NMR (DMSO-d_6_, 100 MHz) *δ* 144.1 (C-2), 137.8 (C-3), 175.8 (C-4), 160.8 (C-5), 98.4 (C-6), 164.4 (C-7), 93.6 (C-8), 156.0 (C-9), 103.2 (C-10), 133.6 (C-1'), 47.0 (C-2'), 96.9 (C-3'), 201.4 (C-4'), 54.3 (C-5'), 130.9 (C-6'), 132.9 (C-1"), 112.5 (C-2"), 147.2 (C-3"), 145.2 (C-4"), 115.1 (C-5"), 120.2 (C-6"), 45.7 (C-7"), 44.0 (C-8"), 72.8 (C-9"), 55.3 (3"-OCH_3_).

### Silyamandin (11)

Colourless prism. ${\left[\alpha \right]}_{D}^{20}$ –42.8 (*c* 0.96, acetone). ^1^H NMR (DMSO-d_6_, 600 MHz) *δ* 12.37 (1H, br s, 4'-COOH), 11.79 (1H, s, 5-OH), 10.90 (1H, s, 7-OH), 8.85 (1H, s, 4"-OH), 6.90 (1H, d, *J* = 1.4 Hz, H-2''), 6.69 (1H, d, *J* = 8.0 Hz, H-5''), 6.66 (1H, dd, *J* = 8.0, 1.4 Hz, H-6''), 6.15 (1H, d, *J* = 5.0 Hz, 3-OH), 6.11 (1H, br s, H-6'), 5.94 (1H, d, *J* = 2.0 Hz, H-8), 5.93 (1H, d, *J* = 2.0 Hz, H-6), 5.12 (1H, d, *J* = 10.4 Hz, H-2), 4.49 (1H, d, *J* = 10.4, 5.0 Hz, H-3), 4.21 (1H, dd, *J* = 9.3, 5.5 Hz, H-9"a), 3.83 (1H, d, *J* = 7.1 Hz, H-2'), 3.76 (1H, d, *J* = 9.3 Hz, H-9"b), 3.75 (3H, s, 3''-OCH_3_), 3.45 (1H, d, *J* = 11.0 Hz, H-5'), 2.94 (1H, m, H-8"), 2.62 (1H, dd, *J* = 12.4, 11.0 Hz, H-7''). ^13^ C NMR (DMSO-d_6_, 150 MHz) *δ* 79.9 (C-2), 73.0 (C-3), 196.8 (C-4), 163.3 (C-5), 95.1 (C-6), 166.9 (C-7), 96.2 (C-8), 162.3 (C-9), 100.1 (C-10), 131.5 (C-1'), 42.1 (C-2'), 175.6 (C-3'), 173.5 (C-4'), 48.8 (C-5'), 125.7 (C-6'), 131.3 (C-1"), 112.1 (C-2"), 147.6 (C-3"), 145.5 (C-4"), 115.2 (C-5"), 121.3 (C-6"), 41.7 (C-7"), 39.3 (C-8"), 68.6 (C-9"), 55.6 (3"-OCH_3_).

### PTP1B inhibitory activity assay

PTP1B inhibitory activity was measured using *p*-NPP as the substrate. A mixture consisting of *p*-NPP and PTP1B in a buffer containing 0.06 M citrate (pH 6.0), 0.1 M NaCl, 1 mM EDTA, and 1 mM DTT with or without a tested compound solution (prepared in the above buffer solution containing 30% dimethyl sulfoxide), was incubated at 37 °C for 30 min. The substrate was used at a concentration of 4 mM. The reaction was terminated by adding 20 µL of 10 M NaOH. The reaction mixture was blended by a microplate mixer for 5 min and the amount of produced *p*-nitrophenol was tested by measuring the absorbance at 405 nm. The blank was measured in the same way except adding a buffer solution instead of the enzyme. The inhibitory activities were further measured at a number of stepwise gradient of concentrations ranging from 100 µM to 0.01 µM to obtain the IC_50_ values by regression analysis. Ursolic acid was used as the positive control due to the commercial availability, similar inhibitory potency, and standardised bioassay process in our lab. All compounds used for bioassay were confirmed the purity >98% by HPLC-PDA and ^1^H NMR spectroscopic analyses.

### TCPTP, VHR, SHP-1, and SHP-2 inhibitory activity assay

TCPTP, VHR, SHP-1, and SHP-2 inhibitory activities were also measured using *p*-NPP as the substrate. For VHR inhibitory activity assay, buffer (pH 7.0) was the same as PTP1B inhibitory activity assay. For TCPTP, SHP-1 and SHP-2 assay, buffer (pH 7.0) was prepared using 25 mM Tris/HCl, 50 mM NaCl, 2 mM EDTA, 5 mM DTT, 0.01% Brij35, and 1 mg/mL bovine serum albumin. The substrate was used at concentrations of 8 mM for TCPTP, VHR, and SHP-2, and 16 mM for SHP-1. The other method was same as PTP1B inhibitory activity assay.

### Molecular docking simulation

Docking experiment was carried out by using a software Biovia Discovery Studio 4.5 (Accelrys Inc., San Diego, CA). The stable structures of compounds were prepared by a standard dynamics cascade. The X-ray crystal structure of PTP1B (PDB code: 1T48, residues 1-283, and 290-298) was obtained from a protein data bank (http://www.rcsb.org). The residues 284–289 were built using the closed form PTP1B crystal structure (PDB code: 5KA9 (residues 1–294, including α7 helix)) to generate PTP1B_1-298_ structure. Docking simulation was carried out using CHARMm-based DOCKER (CDOCKER). The docking results differing by <2.0 Å on the basis of a positional root mean square deviation (RMSD) were clustered together and were ranked on the basis of free binding energy. All other parameters were maintained as default.

## Result and discussion

### Extraction, isolation, and structural identification

**Figure 1.**
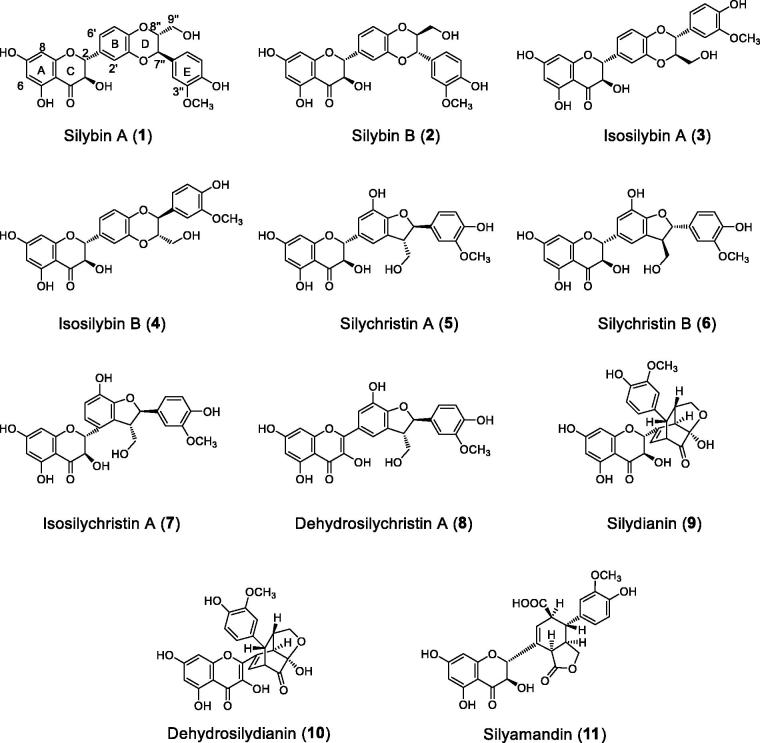
Structures of compounds **1**–**11**.

PTP1B inhibitory assay was used for screening. An extract from the seeds of *S. marianum* was screened as PTP1B inhibitory active. The EtOAc fraction, showing potent inhibitory activity, was subjected to a bioassay-guided fractionation, and afforded two bioactive fractions E and G (IC_50_ = 27.2 and 38.4 µg/mL). Further chromatographic separation led to the isolation of eleven flavonolignans, namely, silybin A (**1**)^23^, silybin B (**2**)^23^, isosilybin A (**3**)^23^, isosilybin B (**4**)^23^, silychristin A (**5**)^24^, silychristin B (**6**)^24^, isosilychristin A (**7**)^24^, dehydrosilychristin A (**8**)^24^, silydianin (**9**)^25^, dehydrosilydianin (**10**)^26^, and silyamandin (**11**)^27^ (Figure 1). The chemical structures were determined by spectroscopic analyses and comparison with those in literatures.

### PTP1B inhibition

The PTP1B inhibitory activities of compounds **1–11** were evaluated. A known PTP1B inhibitor, ursolic acid, was used as the positive control (IC_50_ = 3.52 µM). As the result, nine compounds (**1**–**8** and **11**) inhibited PTP1B in a concentration-dependent manner, and their IC_50_ values were determined by regression analysis (Table 1). Among them, compounds **1** and **4** showed the most potent PTP1B inhibitory activity with IC_50_ values of 1.54 and 1.37 µM, respectively. The stronger inhibitory activity of **1** than **2** (IC_50_ = 5.65 µM), and **4** than **3** (IC_50_ = 2.65 µM), suggesting that the absolute configurations at C-7" and C-8" are important for inhibitory activity. Compound **1** showed more potent activity than **5** (IC_50_ = 5.58 µM), suggesting that the B ring substituents pattern greatly affected the activity. The activities of compounds **5** (IC_50_ = 5.58 µM), **7** (IC_50_ = 6.55 µM), and **8** (IC_50_ = 6.55 µM) were almost same, indicating that the C ring double bond between C-2 and C-3 did not affect the activity. Furthermore, silydianin type flavanolignans **9** and **10** exhibited no activity (IC_50_ > 50 µM).

**Table 1.** Inhibition effects of compounds **1**–**11**, and ursolic acid against PTP1B.

Compound	IC (μM)^a^	Inhibition type	*K*_i_ (μM)
**1**	1.54 ± 0.22	Non-competitive	1.25
**2**	5.65 ± 0.20	Non-competitive	4.05
**3**	2.65 ± 0.13	Non-competitive	2.25
**4**	1.37 ± 0.22	Non-competitive	1.03
**5**	5.58 ± 0.33	Non-competitive	3.95
**6**	23.87 ± 2.25		
**7**	6.55 ± 0.09		
**8**	7.18 ± 0.12		
**9**	NA		
**10**	NA		
**11**	17.38 ± 0.67		
Ursolic acid^b^	3.52 ± 0.10		

NA, no activity >50 μM.

^a^Values are the means ± SD, *n* = 3.

^b^Positive control.

### Kinetic analysis

To elucidate the inhibition mode, the inhibition kinetics of compounds **1**–**5** were analyzed by the Lineweaver–Burk method with various substrate concentrations of *p*-NPP (4, 8, 16 mM). The initial reaction velocities were measured with and without the inhibitor. The *V*_max_ values increased in a dose-dependent manner without changing the *K*_m_ value, indicating that they inhibited PTP1B activity by a non-competitive mechanism (Figure 2). The secondary plot of slope from the Lineweaver–Burk plot on the *y*-axis against the concentration of the compound on the *x*-axis, obtained the quadratic-like curves, exhibited a good linear plot. Dissociation constant (*K*_i_) values of compounds **1**–**5** were calculated as 1.25, 4.05, 2.25, 1.03, and 3.95 µM, respectively.

Figure 2.(A) Lineweaver–Burk plots of compounds **1**–**5**. The final concentration of compounds **1**–**5** was as follows: **1**; 0, 1.0, 2.0, and 3.0 μM, **2**; 0, 4.0, 6.0, and 8.0 μM, **3**; 0, 1.3, 2.5, and 3.0 μM, **4**; 0, 1.0, 2.0, and 3.0 μM, and **5**; 0, 4.0, 6.0, and 8.0 μM. (B) The second plots of A.

**Figure:**
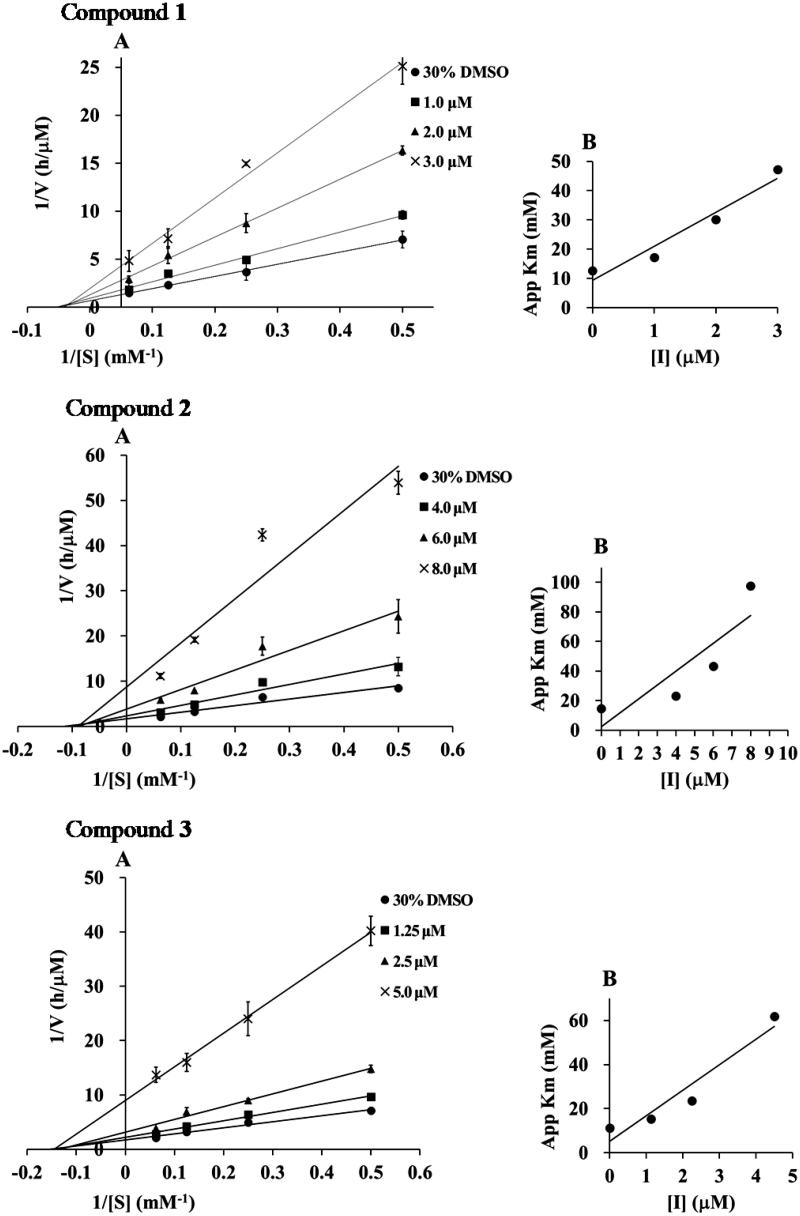

**Figure:**
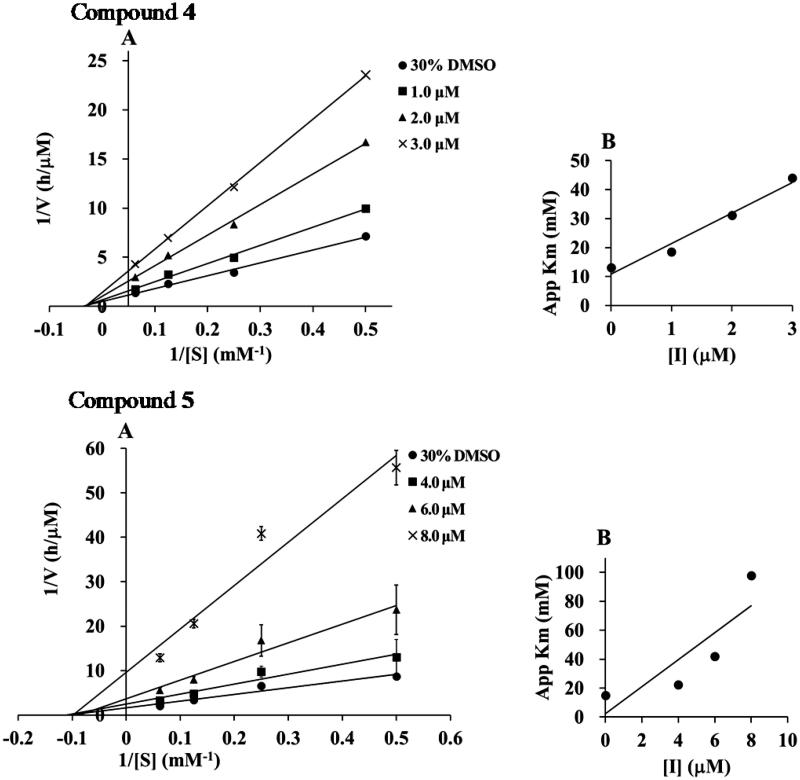

### Docking simulation

**Figure 3.**
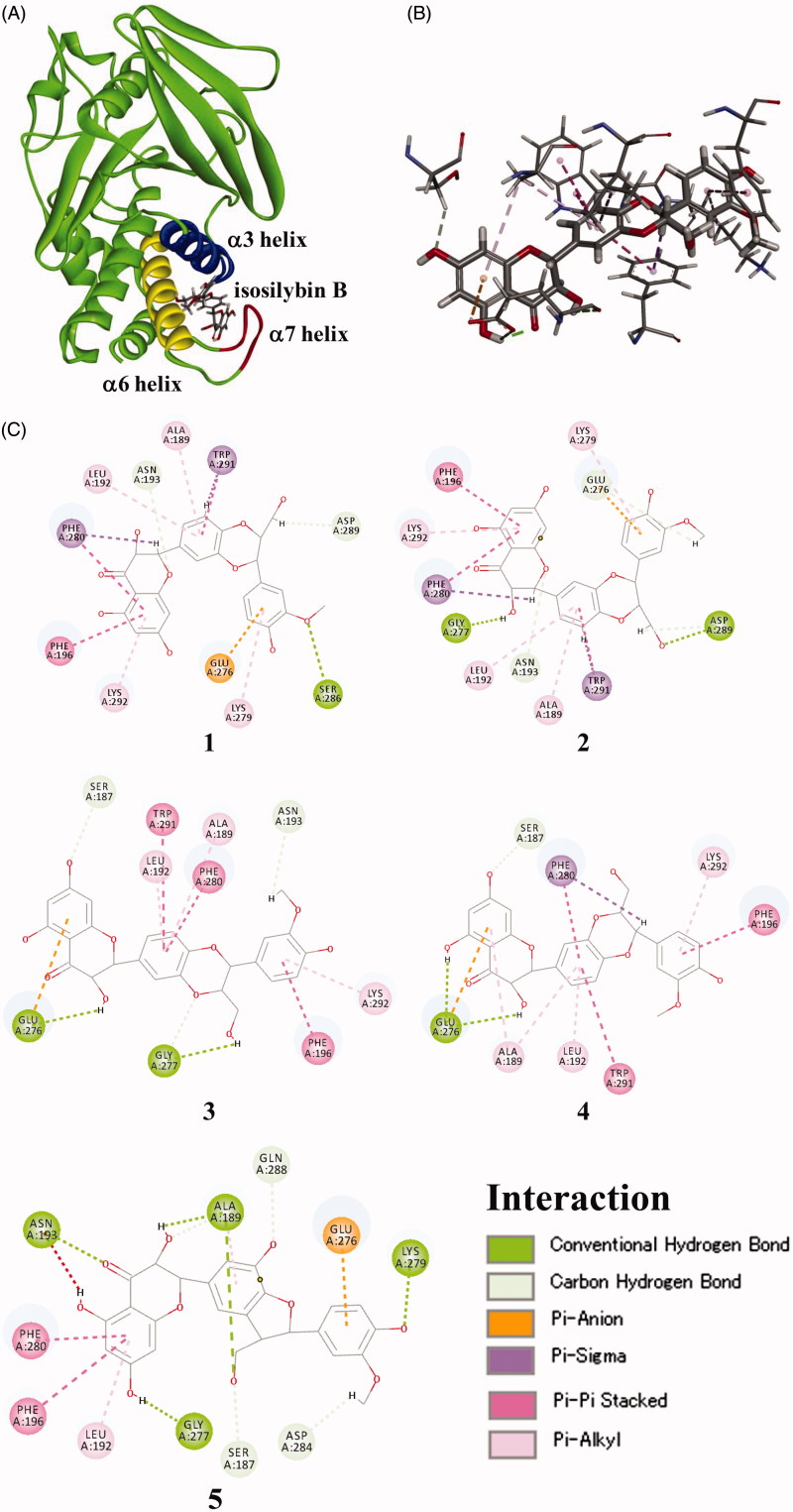
Docked molecular model of compounds **1**–**5** in the allosteric site of the PTP1B enzyme. (A) 3D docking molecules of compound **4** of the PTP1B (PDB code: 1T48): the blue line represents the α3 helix, the yellow line represents the α6 helix, and the red line represents the α7 helix. (B) Binding interactions of compound **4**. Hydrogen bond interactions were shown as green dashed lines, carbon–hydrogen bond interactions were shown as thin green dashed lines, pi–anion interaction was shown as orange dashed line, pi–sigma interaction was shown as purple dashed line, pi–pi stacked interaction was shown as pink dashed line, and pi–alkyl interaction was shown as thin pink dashed line (for interpretation of the references to colour in this figure legend, the reader is referred to the web version of this article). (C) 2D diagram of the interaction of compounds **1**–**5**.

As compounds **1**–**5** were non-competitive type PTP1B inhibitors, their binding mode to the allosteric site of PTP1B was investigated by a docking simulation using Biovia Discovery Studio 4.5^19^. Preferred coordination mode of compounds **1**–**5** with the allosteric site of PTP1B is shown (Figure 3). The binding energy of compounds **1**–**5** for the docking experiment was calculated to be 32.5, 25.9, 31.7, 39.2, and 22.5 kcal/mol, respectively, suggesting that the docking energy was proportional to IC_50_ values.

Compounds **1**–**5** interacted with the amino acid residues belonging to α3, α6, and α7 helix of PTP1B (Figure 3). Four amino acid residues, namely, Ala189, Leu192, and Phe196 in α3 helix, and Glu276 in α6 helix of PTP1B seem to be important for the inhibitory activity of flavonolignans, as they were commonly binding to all five compounds. Compounds **1** and **2**, which are different from their structures only by the configurations at C-7" and C-8", were binding to PTP1B similarly. The fact that compound **1** showing more potent inhibitory activity than **2**, maybe interpretable by a hydrogen bond between 3''-OCH_3_ and Ser286, which was only observed in **1**. In contrast, compounds **3** and **4** are also a pair of diastereomers at C-7" and C-8", and they are binding to PTP1B similarly too. The structure of **3** was in favour of PTP1B inhibition due to the multiple interactions of Glu276 and Ala189 with **3**. In a word, compounds **1**–**5** blocked the interaction between α7 and α3–α6, and prevented the closure of catalytic WPD loop, which is in agreement of the known allosteric PTP1B inhibitors^28^.

### PTP selectivity

Because of the high structural similarity of the catalytic centre among the family of protein tyrosine phosphatases^29^, the inhibitory selectivity is an important evaluation index for development of PTP1B inhibitors. Four non-receptor-like and cytosolic PTPase homologous protein tyrosine phosphatases, namely, TCPTP, VHR, SHP-1, and SHP-2 were selected for comparison the PTPs inhibitory activities. Compounds **1**–**5** were evaluated their inhibitory activity against five PTPs at their PTP1B completely inhibitory concentrations, respectively. As the results, compounds **1**–**5** share the similar profile of PTP inhibitory selectivity (Table 2). Namely, when compounds **1**–**5** completely inhibited PTP1B, they moderately inhibited VHR (inhibition rate: 59.2–67.5%), and SHP-2 (32.7–50.1%), and weakly inhibited TCPTP (15.7–32.2%) and SHP-1 (22.9–33.4%). Among the five compounds, **1** showed the best inhibitory selectivity between PPTP1B and other PTPs but still, need further improvement.

**Table 2.** Inhibition rate (%) of compounds **1**–**5** against PTP1B, TCPTP, VHR, SHP-1, and SHP-2.

	Compound **1**	Compound **2**	Compound **3**
PTP	Inhibition rate (%)^a^	Inhibition rate (%)^a^	Inhibition rate (%)^a^
PTP1B	90.3 ± 0.9	89.0 ± 2.1	90.7 ± 1.1
TCPTP	15.7 ± 1.9	32.1 ± 2.3	27.5 ± 0.8
VHR	60.6 ± 2.2	65.6 ± 4.2	59.2 ± 2.4
SHP-1	33.4 ± 0.4	34.4 ± 1.9	30.6 ± 1.8
SHP-2	32.7 ± 1.8	37.5 ± 2.2	49.0 ± 2.1
	Compound **4**	Compound **5**	
PTP	Inhibition rate (%)^a^	Inhibition rate (%)^a^	
PTP1B	92.1 ± 1.7	89.2 ± 2.6	
TCPTP	16.4 ± 1.5	32.2 ± 1.9	
VHR	61.4 ± 3.0	67.5 ± 1.4	
SHP-1	22.9 ± 2.1	25.9 ± 1.4	
SHP-2	50.1 ± 1.5	48.6 ± 1.9	

^a^Inhibition rate (%) is mean ± SD from three separate experiments at sample final concentrations: Compounds **1** and **4**: 4.0 μM, **2**: 12 μM, **3**: 6.0 μM, **5**: 20 μM.

## Conclusions

In conclusion, nine flavonolignans from seeds of *S. marianum* were identified as a novel class of novel natural PTP1B inhibitors. Among them, compounds **1**–**5**, which showed potent inhibitory activities (IC_50_ 1.37–5.65 µM), were demonstrated to be non-competitive PTP1B inhibitors, and with inhibitory selectivity between PTP1B and other PTPs. Docking analysis supported the results of PTP1B inhibitory activity assay and kinetics analysis. Flavonolignans from seeds of *S. marianum* have been demonstrated to have beneficial effects against diabetic mellitus. The antidiabetic effect of silybin A (**1**) was attributed to inhibition of gluconeogenesis in the liver and decrease of glucose-6 phosphatase activity^30^. In addition, isosilybin (**3**) has been identified as an agonist of peroxisome proliferator-activated receptor γ^14^. Our investigation demonstrated that these PTP1B inhibitory compounds could have potential as lead compounds for further anti-diabetes drug developments.

## References

[1] Brown-Shimer S, Johnson KA, Lawrence JB, et al. Molecular cloning and chromosome mapping of the human gene encoding protein phosphotyrosyl phosphatase 1B. Proc Natl Acad Sci USA 1990;87:5148–52. 2164224 10.1073/pnas.87.13.5148 PMC54279

[2] Kennedy BP Role of protein tyrosine phosphatase-1B in diabetes and obesity. Biomed Pharmacother 1999;53:466–70. 10665340 10.1016/s0753-3322(00)88105-6

[3] Klaman LD, Boss O, Peroni OD, et al. Increased energy expenditure, decreased adiposity, and tissue-specific insulin sensitivity in protein-tyrosine phosphatase 1B-deficient mice. Mol Cell Biol 2000;20:5479–89. 10891488 10.1128/mcb.20.15.5479-5489.2000 PMC85999

[4] Zhang S, Zhang ZY PTP1B as a drug target: recent developments in PTP1B inhibitor discovery. Drug Discov Today 2007;12:373–81. 17467573 10.1016/j.drudis.2007.03.011

[5] Bentires AM, Neel BG Protein-tyrosine phosphatase 1B is required for HER2/Neu-induced breast cancer. Cancer Res 2007;67:2420–4. 17347513 10.1158/0008-5472.CAN-06-4610

[6] Erbe DV, Wang S, Zhang YL, et al. Ertiprotafib improves glycemic control and lowers lipids via multiple mechanisms. Mol Pharmacol 2005;67:69–77. 15475571 10.1124/mol.104.005553

[7] Lantz KA, Hart SG, Planey SL, et al. Inhibition of PTP1B by trodusquemine (MSI-1436) causes fat-specific weight loss in diet-induced obese mice. Obesity (Silver Spring) 2010;18:1516–23. 20075852 10.1038/oby.2009.444

[8] He RJ, Yu ZH, Zhang RY, Zhang ZY Protein tyrosine phosphatases as potential therapeutic targets. Acta Pharmacol Sin 2014;35:1227–46. 25220640 10.1038/aps.2014.80 PMC4186993

[9] Combs AP Recent advances in the discovery of competitive protein tyrosine phosphatase 1B inhibitors for the treatment of diabetes, obesity, and cancer. J Med Chem 2010;53:2333–44. 20000419 10.1021/jm901090b

[10] Biagi M, Pecorari R, Appendino G, et al. Herbal products in Italy: the thin line between phytotherapy, nutrition and parapharmaceuticals; a normative overview of the fastest growing market in Europe. Pharmaceuticals 2016;9:65–74. 10.3390/ph9040065 PMC5198040 27801865

[11] Hackett ES, Twedt DC, Gustafson DL Milk thistle and its derivative compounds: a review of opportunities for treatment of liver disease. J Vet Intern Med 2013;27:10–6. 23140176 10.1111/jvim.12002

[12] Schümann J, Prockl J, Kiemer AK, et al. Silibinin protects mice from T cell-dependent liver injury. J Hepatol 2003;39:333–40. 12927918 10.1016/s0168-8278(03)00239-3

[13] Trappoliere M, Caligiuri A, Schmid M, et al Silybin, a component of sylimarin, exerts anti-inflammatory and anti-fibrogenic effects on human hepatic stellate cells. J Hepatol 2009;50:1102–11. 19398228 10.1016/j.jhep.2009.02.023

[14] Pferschy-Wenzig EM, Atanasov AG, Malainer C, et al. Identification of isosilybin A from milk thistle seeds as an agonist of peroxisome proliferator-activated receptor gamma. J Nat Prod 2014;77:842–7. 24597776 10.1021/np400943b PMC4003856

[15] Voroneanu L, Nistor I, Dumea R, et al. Silymarin in type 2 diabetes mellitus: a systematic review and meta-analysis of randomized controlled trials. J Diabetes Res 2016;2016:1. 10.1155/2016/5147468 PMC4908257 27340676

[16] Soto C, Raya L, Juárez J, et al. Effect of silymarin in Pdx-1 expression and the proliferation of pancreatic β-cells in a pancreatectomy model. Phytomedicine 2014;21:233–9. 24176839 10.1016/j.phymed.2013.09.008

[17] Ebrahimpour koujan S, Gargari BP, Mobasseri M, et al. Effects of *Silybum marianum* (L.) Gaertn. (silymarin) extract supplementation on antioxidant status and hs-CRP in patients with type 2 diabetes mellitus: a randomized, triple-blind, placebo-controlled clinical trial. Phytomedicine 2015;22:290–6. 25765835 10.1016/j.phymed.2014.12.010

[18] Onoda T, Li W, Sasaki T, et al. Identification and evaluation of magnolol and chrysophanol as the principle protein tyrosine phosphatase-1B inhibitory compounds in a Kampo medicine, Masiningan. J Ethnopharmacol 2016;186:84–90. 27049294 10.1016/j.jep.2016.03.063

[19] Sasaki T, Li W, Higai K, Koike K Canthinone alkaloids are novel protein tyrosine phosphatase 1B inhibitors. Bioorg Med Chem Lett 2015;25:1979–81. 25819098 10.1016/j.bmcl.2015.03.014

[20] Sasaki T, Li W, Higai K, et al. Protein tyrosine phosphatase 1B inhibitory activity of lavandulyl flavonoids from roots of *Sophora flavescens* . Planta Med 2014;80:557–60. 24782228 10.1055/s-0034-1368400

[21] Li W, Li S, Higai K, et al. Evaluation of licorice flavonoids as protein tyrosine phosphatase 1B inhibitors. Bioorg Med Chem Lett 2013;23:5836–9. 24047800 10.1016/j.bmcl.2013.08.102

[22] Sasaki T, Li W, Morimura H, et al. Chemical constituents from *Sambucus adnata* and their protein-tyrosine phosphatase 1B inhibitory activities. Chem Pharm Bull 2011;59:1396–9. 22041077 10.1248/cpb.59.1396

[23] Lee DYW, Liu Y Molecular structure and stereochemistry of silybin A, silybin B, isosilybin A, and isosilybin B, isolated from *Silybum marianum* (milk thistle). J Nat Prod 2003;66:1171–4. 14510591 10.1021/np030163b

[24] Wang C, Zhang X, Wei P, et al. Chemical constituents from *Inula wissmanniana* and their anti-inflammatory activities. Arch Pharm Res 2013;36:1516–24. 23703255 10.1007/s12272-013-0143-1

[25] Kim NC, Graf TN, Sparacino CM, et al. Complete isolation and characterization of silybins and isosilybins from milk thistle (*Silybum marianum*). Org Biomol Chem 2003;1:1684–9. 12926355 10.1039/b300099k

[26] Pyszková M, Biler M, Biedermann D, et al. Flavonolignan 2, 3-dehydroderivatives: preparation, antiradical and cytoprotective activity. Free Radic Biol Med 2016;90:114–25. 26582372 10.1016/j.freeradbiomed.2015.11.014

[27] MacKinnon SL, Hodder M, Craft C, Simmons-Boyce J Silyamandin, a new flavonolignan isolated from milk thistle tinctures. Planta Med 2007;73:1214–6. 17823870 10.1055/s-2007-981595

[28] Wiesmann C, Barr KJ, Kung J, et al. Allosteric inhibition of protein tyrosine phosphatase 1B. Nat Struct Mol Biol 2004;11:730–7. 15258570 10.1038/nsmb803

[29] Tabernero L, Aricescu AR, Jones EY, Szedlacsek SE Protein tyrosine phosphatases: structure-function relationships. FEBS J 2008;275:867–82. 18298793 10.1111/j.1742-4658.2008.06251.x

[30] Guigas B, Naboulsi R, Villanueva GR, et al. The flavonoid silibinin decreases glucose-6-phosphate hydrolysis in perifused rat hepatocytes by an inhibitory effect on glucose-6-phosphatase. Cell Physiol Biochem 2007;20:925–34. 17982275 10.1159/000110453

